# Supplementary material for: Population structure and pharmacogenomic risk stratification in the United States
Source: BMC Biol. 2020 Oct 13;18:140. doi: 10.1186/s12915-020-00875-4 (PMC7557099; doi:10.1186/s12915-020-00875-4)
Supplement: Supplementary file 1 — Additional file 1. Table S1, Figures S1–4. [file 12915_2020_875_MOESM1_ESM.pdf]

Supplementary Material

Population structure and pharmacogenomic risk stratification in the United States

Shashwat Deepali Nagar, Andrew B. Conley, and I. King Jordan

Table of Contents

Supplementary Table S1. Global reference populations used for genetic ancestry inference. 2

Supplementary Figure 1. Permutation analysis to evaluate the stability of *k*-means genetic ancestry (GA) clusters. 3

Supplementary Figure 2. Comparison of self-identified race/ethnicity (SIRE) versus genetic ancestry (GA) groups in the US. 4

Supplementary Figure 3. Correspondence between self-identified race/ethnicity (SIRE) versus genetic ancestry (GA) groups in the US. 5

Supplementary Figure 4. Pharmacogenomic variation in the US: genetic ancestry (GA). 6

Supplementary Figure 5. Distributions of F_{ST} for PGx variants. 7

Supplementary Table S1. **Global reference populations used for genetic ancestry inference.**

Population[1]	N[2]	Continental ancestry[3]	Source[4]
African Caribbean in Barbados	94	African	1KGP
Algonquin	5	Native American	Reich et al.
Americans of African ancestry from SW USA	51	African	1KGP
Utah Residents with Northern and Western European Ancestry	99	European	1KGP
Chipewyan	13	Native American	Reich et al.
Cree	4	Native American	Reich et al.
Finnish in Finland	99	European	1KGP
French	28	European	HGDP
British in England and Scotland	91	European	1KGP
Iberian Population in Spain	107	European	1KGP
Mixe	17	Native American	Reich et al.
Mixtec	5	Native American	Reich et al.
Ojibwa	5	Native American	Reich et al.
Orcadian	15	European	HGDP
Piapoco	7	Native American	Reich et al.
Pima	14	Native American	HGDP
Russian	25	European	HGDP
Sardinian	28	European	HGDP
Tepehuano	25	Native American	Reich et al.
Teribe	3	Native American	Reich et al.
Ticuna	6	Native American	Reich et al.
Toscani in Italia	107	European	1KGP

[1]Population name

[2]Number of samples

[3]Population continental ancestry

[4]Data source: 1000 Genomes Project (1KGP), Human Genome Diversity Project (HGDP), Collection of Native American Samples (Reich et al. Nature 2012 488: 370).

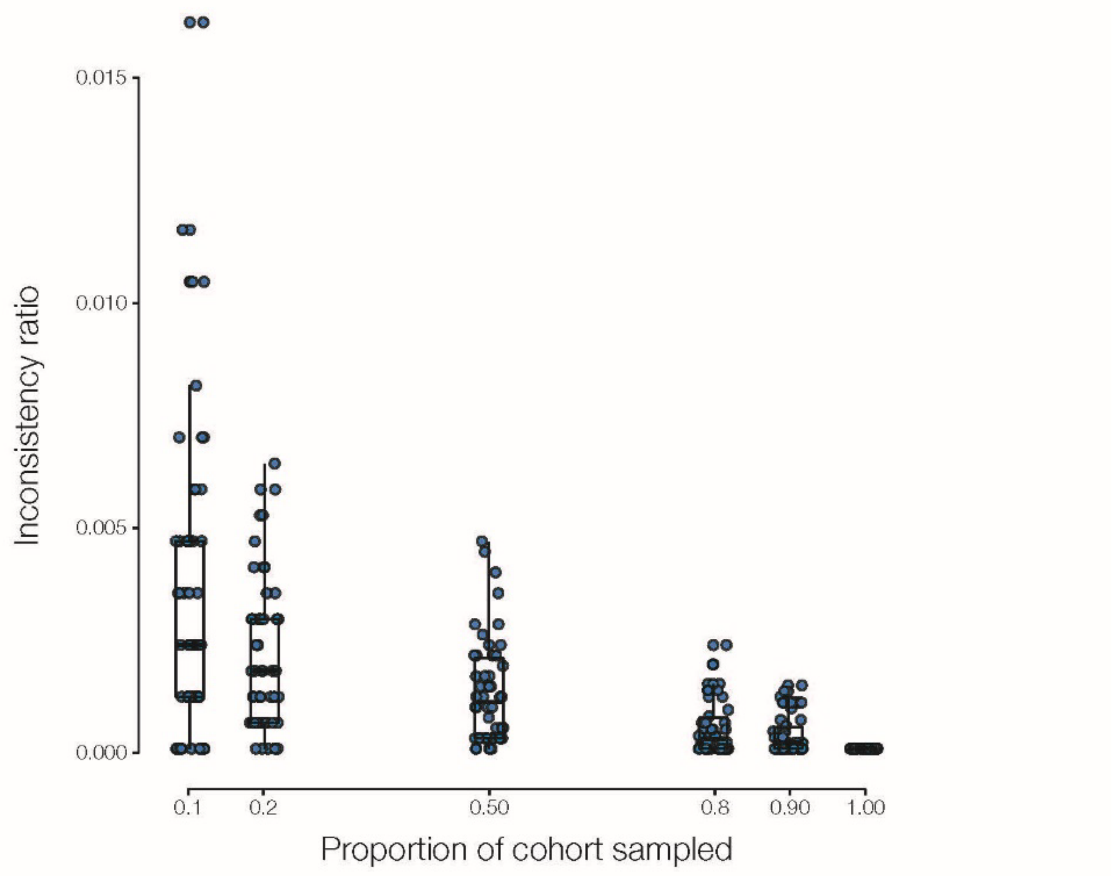

Supplementary Figure 1. **Permutation analysis to evaluate the stability of *k*-means genetic ancestry (GA) clusters.** The HRS cohort was randomly sampled at different proportions, where the proportion of the cohort sampled = the number of participants in the random sample / the total number of participants in the cohort. For each random sample, *k*-means clustering was run 50 times and an inconsistency ratio was calculated for each independent run, where the inconsistency ratio is the number of mismatches between the random sample group assignments / the number of participants in the random sample. In other words, the inconsistency ratio measures the error in *k*-means cluster assignments due to sampling bias. As can be expected, error is higher for smaller random cohort proportions and decreases monotonically as the proportion of the random cohorts increases. Nevertheless, the error level, even at the smallest sampling proportions, is extremely low. The mean error at a sampling proportion of 0.1 is 0.4%, and when the entire cohort is sampled (i.e. cohort proportion=1) *k*-means clustering is 100% consistent.

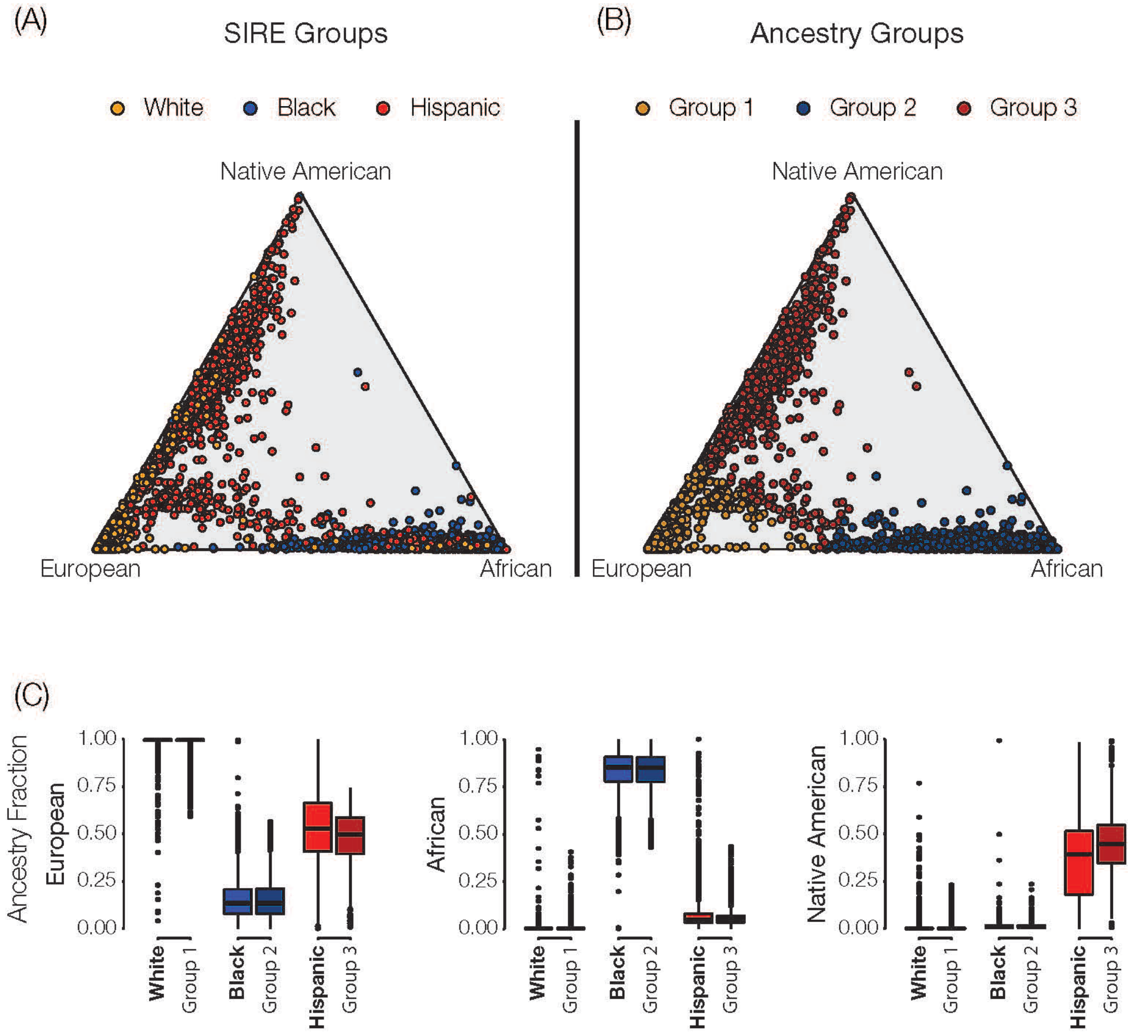

Supplementary Figure 2. **Comparison of self-identified race/ethnicity (SIRE) versus genetic ancestry (GA) groups in the US.** Ternary plots showing the relative continental ancestry fractions for HRS participants are shown with individuals color coded by SIRE (A) or genetic ancestry (B). SIRE and their corresponding GA groups are coded as White/Group 1 (orange), Black/Group 2 (blue), and Hispanic/Group 3 (red). (C) Distributions of continental ancestry fractions – European, African, and Native American – for HRS participants are shown corresponding SIRE and GA groups.

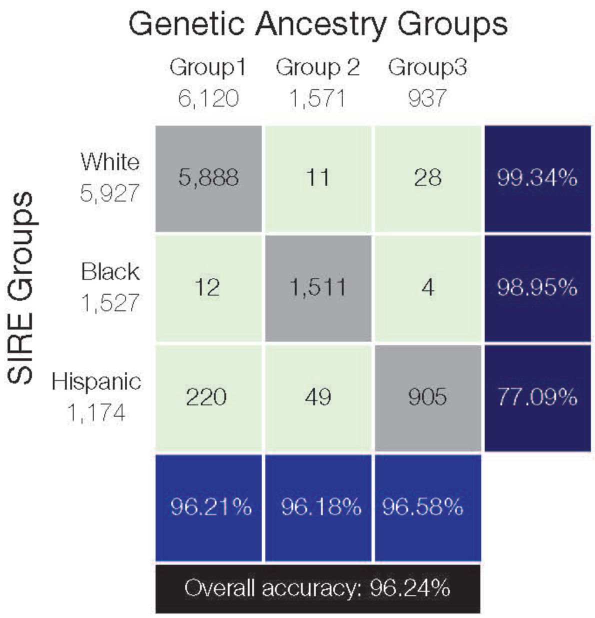

Supplementary Figure 3. **Correspondence between self-identified race/ethnicity (SIRE) versus genetic ancestry (GA) groups in the US.** Numbers of HRS participants that fall into each combination of SIRE and GA groups is shown along with the percentage correspondence. Individual percent correspondence values are calculated as the number of individuals along the diagonal, i.e. that fall into the corresponding SIRE and GA groups, divided by the total number of individuals in each SIRE group (right) or each GA group (bottom), times 100. The overall percent correspondence is calculated as the number of individuals along the diagonal divided by the total number of individuals in the HRS cohort, times 100.

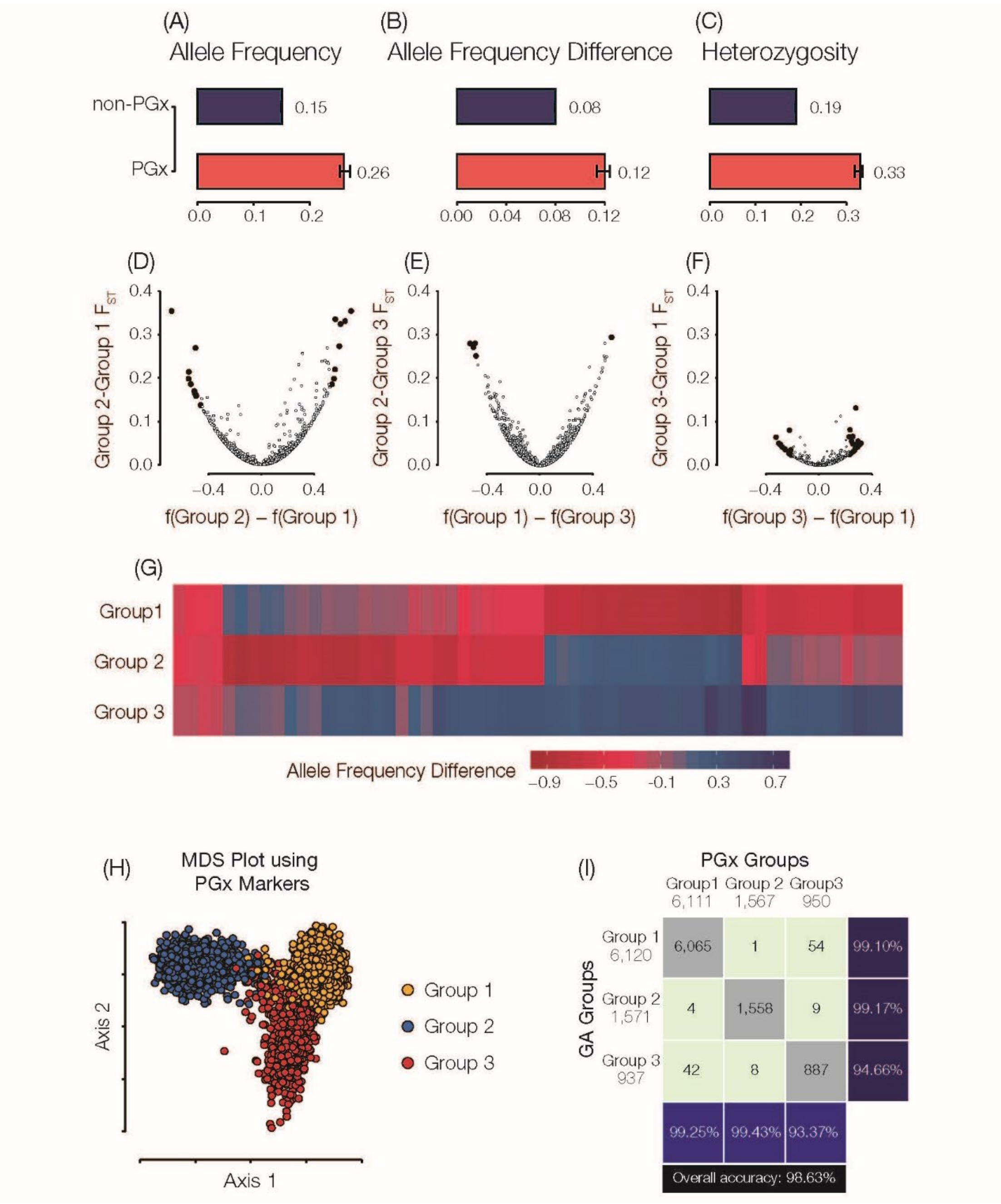

Supplementary Figure 4. **Pharmacogenomic variation in the US: genetic ancestry (GA).** Data shown here correspond to GA groups; analogous results for SIRE groups shown in Figure 2. Genome-wide average allele frequencies (A), group-specific allele frequency differences (B), and heterozygosity fractions (C) are shown for PGx variants (red) compared to non-PGx variants (blue). (D-F) Fixation index (F_{ST}; y-axis) and allele frequency differences (x-axis) for pairs of GA groups. Statistically significant PGx allele frequency differences are highlighted in black. (G) Heatmap showing group-specific allele frequencies for significantly diverged PGx variants. (H) Multi-dimensional scaling (MDS) plot showing the relationship among individual genomes as measured by PGx variants alone. Each dot is an individual HRS participant genome, and genomes are color-coded by participants GA groups. (I) The correspondence between GA groups and PGx groups defined by K-means clustering on the results of the MDS analysis.

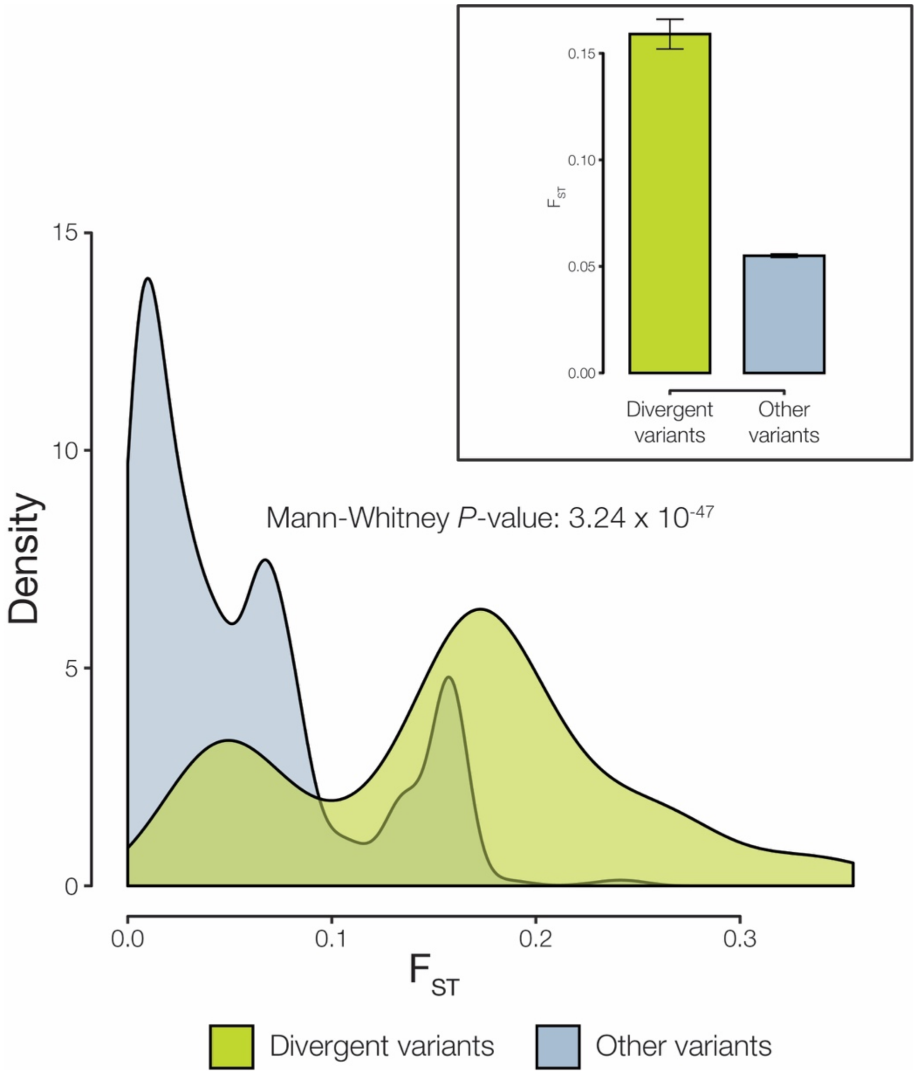

Supplementary Figure 5. **Distributions of F_{ST} for PGx variants.** Distribution for the 82 significantly diverged PGx variants shown in Figure 2G (yellow) and all other PGx variants (blue). The inset shows mean and standard error F_{ST} values. Average divergent PGx variant F_{ST} = 0.15 ± 0.007 compared to an average F_{ST} = 0.05 ± 0.0008 for the other variants (Mann Whitney U=408,550 P=3.2 × 10^{-47}).
